# Unusual intron in the second exon of a Type III polyketide synthase gene of *Alpinia calcarata* Rosc.

**DOI:** 10.1590/S1415-47572010000100024

**Published:** 2010-03-01

**Authors:** Edayileveettil K Radhakrishnan, Rintu T Varghese, Soniya E Vasudevan

**Affiliations:** Plant Molecular Biology Division, Rajiv Gandhi Centre for Biotechnology, Thiruvananthapuram, KeralaIndia

**Keywords:** *Alpinia calcarata*, CHS, Type III polyketide synthase, phylogenetic analysis

## Abstract

Plant phenolic compounds form a valuable resource of secondary metabolites having a broad spectrum of biological activities. Type III polyketide synthases play a key role in the formation of basic structural skeleton of the phenolic compounds. As a group of medicinal plants, PKSs with novel features are expected in the genome of Zingiberaceae. The genomic exploration of PKS in *Alpinia calcarata* conducted in this study identified the presence of an unusual intron at the region forming the second exon of typical PKSs, forming a gateway information of distribution of novel PKSs in Zingiberaceae.

The Type III polyketide synthase (PKS) superfamily of enzymes play an important role in the biosynthesis of phenolic compounds of diverse structure and function in plants (Austin and Noel, 2003). Chalcone synthase is the most common and widely distributed member of the PKS superfamily, consisting of homodimers of a 40-45 kDa polypeptide. In the typical enzymatic reaction, PKS forms the chalcone by stepwise decarboxylative condensation of one coumaroyl CoA molecule with three malonyl CoA moieties, followed by a Claisen type cyclization of the tetraketide product (Ferrer *et al.*, 1999). The amazing biochemical diversity of PKSs is shown by the identification and characterization of expanding members of the family, such as the 2-pyrone synthase, stilbene synthase, benzalacetone synthase, valerophenone synthase, acridon synthase, etc. (Austin and Noel, 2003, Radhakrishnan *et al.*, 2009). But considering the huge metabolic complexity of plants, more members are very likely to occur in plants, especially in medicinal plants.

Polyketide synthases have highly conserved stretches of amino acid residues forming the catalytic pockets, and the residues forming these pockets are located mainly within the second exon (Brand *et al.*, 2006). This facilitates the use of PCR based methods to identify the genomic distribution of PKSs in taxonomically diverse plants. The genomic information derived from such studies can give molecular insights into functional novelties, since the substitution of key amino acid residues may have a tremendous impact on the PKS reaction mechanism. Zingiberaceae plants are well known for their metabolite richness, and the distribution of structurally diverse phenolic compounds across the species reflects the genomic possibly novel PKS forms. So it is very probable that PKSs in Zingiberaceae might have been subject to remarkable genomic changes. In the current study, a PCR based investigation of a PKS gene was conducted in *Alpinia calcarata*, to unveil its genomic features.

Plants of *Alpinia calcarata* Rosc. were obtained from the Kerala Ayurvedic Research Institute, Thiruvananthapuram, Kerala, and were grown and maintained in the experimental plant garden at Rajiv Gandhi Centre for Biotechnology, Thiruvananthapuram, Kerala. Genomic DNA was isolated from young leaves by a modified CTAB protocol (Murray and Thompson, 1980). The quality of the DNA was analyzed by agarose gel electrophoresis and was quantified spectrophotometrically using Biospec-1601, DNA/Protein/Enzymes Analyzer (Shimadzu).

Primers for amplifying the second exon of PKS of *A. calcarata* were designed manually. For this, the PKS gene sequences reported from other plants were retrieved from NCBI and were aligned using the Clustal W program (Thompson *et al.*, 1994). Sequences at the most conserved region were selected for designing the primers GPKSF (5' CCTCGCCAAGGACCTCGCCGAGAACAA-3') and GPKSR (5' CGGACCGAACCCGAAGAGAACGCCCC A-3'). The PCR reaction mix (50 μL) contained 100 ng of genomic DNA, 20 pmol of each primer, 1.5 units of *Taq* DNA polymerase (Promega), 1.5 mM MgCl_2_ and 200 μM of each dNTPs. Amplifications were carried out in a BioRad iCycler using the following conditions: initial denaturation for 5 min at 94 °C, 35 cycles of 94 °C for 30 s, 65 °C for 1 min and 72 °C for 1 min, followed by a final extension at 72 °C for 7 min. The PCR products were analyzed on 2% agarose gel and purified using the GFX gel band purification kit (Amersham). The purified PCR product was ligated into pGEM-T Easy plasmid vector (Promega). The ligation reaction mix (10 μL) contained 1X ligation buffer, 50 ng of PCR product, 50 ng of pGEM-T Easy vector DNA and 3 units of T4 DNA ligase. The ligation was carried out overnight at 4 °C and the product was used for transformation. Positive transformants were selected by blue white screening and colony PCR. Plasmid isolation was carried out from positive colonies by alkaline lysis (Birnboim and Doly, 1979) and sequenced using the Big Dye Terminator Cycle Sequencing Ready Reaction Kit Version.3.1 (Applied Biosystem).

Following BLAST analyses (BLASTN and BLASTX), multiple sequence alignments with selected PKSs were run using the Clustal W program. For this the sequences were first aligned at the amino acid level and subsequently, the nucleic acid sequences were aligned according to the amino acid sequences. Intron prediction was done by using the intron prediction program at NetPlantGene Server.

Phylogenetic analysis was carried out using the Neighbour Joining (NJ) and Maximum Parsimony (MP) methods implemented in MEGA3 (Kumar *et al.*,2004). The intron sequence of AcPKS was removed manually and was used for the phylogenetic analysis along with other sequences. Tree topology robustness was assessed by bootstrap analysis with 1000 resampling replicates for the NJ and MP methods. The accession numbers of the PKS sequences selected for the phylogenetic analysis along with AcPKS are as follows; *Aloe arborescens* PCS (AY823626), *Aloe arborescens* OKS (AY567707), *Arabis alpina* CHS (AF112084), *Arachis hypogaea* RES (DQ124938), *Arabidopsis thaliana* CHS (NM_121396, *Bromheadia finlaysoniana* CHS 3 (AF007097), *Callistephus chinensis* CHS (Z67988), *Camellia sinensis* CHS (D26593*)*, *Cardamine amara* CHS (AF112085), *Daucus carota* CHS (AJ006780), *Dictamnus albus* CHS (AJ850132), *Escherichia coli* fabH (M96793), *Gerbera hybrida* 2-PS (Z38097), *Humulus lupulus* VPS (AB047593), *Hordeum vulgare* CHS (X58339), *Hydrangea macrophylla* CTAS (AB011468), *Hypericum androsaemum* BPS (AF352395), *Ipomoea purpurea* CHS (AB001826), *Ipomoea batatas* CHS (AB037389), *Lilium* hybrid CHS (AF169798), *Medicago sativa* CHS (L02901), *Oryza sativa* CHS (X89859), *Petunia hybrida* CHS (X14593), *Phalaenopsis* sp. ‘pSPORT1' BBS (X79903), *Phaseolus vulgaris* CHS (X06411), *Pinus strobus* CHS (AJ004800), *Pisum sativum* CHS (X63335), *Psilotum nudum* STS (AB022685), *Rheum palmatum* BAS (AF326911), *Rheum palmatum* ALS (AY517486), *Rorippa amphibia* CHS (AF144530), *Ruta graveolens* ACS (AJ297786), *Sorghum bicolor* CHS (AY069951), *Triticum aestivum* CHS (AY286098), *Vitis vinifera* STS (EF192465), *Wachendorfia thyrsiflora* PKS (AY727928), *Zingiber officinale* PKS (DQ486012), *Brassica napus* CHS (AF076333), *Dendrobium nobile* CHS (ABE77392), *Iris germanica* CHS (BAE53636), *Curcuma longa* CURS3 (AB506763), *Curcuma longa* CURS2 (AB506762), *Curcuma longa* CURS1 (AB495007), *Curcuma longa* DCS(AB495006), *Oryza sativa* CUS (AK109558) and *Streptomyces griseus* RppA (AB018074).

Use of PKS specific primers for the PCR resulted in the formation of a 703 bp product from genomic DNA of *Alpinia calcarata* (Figure 1). This amplicon size was higher than expected because the primers were selected to give a 600 bp product. This was also expected from our previous experiments on the PKS gene family in *Zingiber officinale*. So the genomic DNA of *Z. officinale* was used as a control. In the BLAST analysis, the nucleotide sequence of *A. calcarata* PKS identified in this study (AcPKS, GenBank accession number EU399815) showed a highest identity of 87% to the PKS/CHS of *Zingiber officinale*, with the core fragment coinciding with the region between 546-1101 bp of typical PKS/CHS. Type III PKSs in plants generally have a size of 1170 bp, coding for 390 amino acids. The most characteristic feature of the core region is its location in the second exon, which contains most of the residues of the catalytic region. Comparative analysis of PKS/CHS sequences from all the plants revealed that all of them have only one intron, except for *Antirrhinum majus* (Sommer and Saedler, 1986). The first exon of CHS encodes 37-64 amino acid residues and the second exon codes for about 340 amino acid residues. Intron size can, however, vary from less than 100 bp to several kilobases (Oberholzer *et al.*, 2000). Due to the unusual size of AcPKS, it formed a gap of 93 bp in the BLAST analysis, making the genomic data more interesting. This was also reflected in the multiple sequence alignment confirming that the extra sequence is specific to AcPKS and is very unusual. Intron prediction analysis identified a potential donor splice site at 217 bp and an acceptor splice site at the 310 bp position in AcPKS (Figure 1). The intron was found to have a size of 93 bp, and its presence at this region is very uncommon or even novel. The intron sequence was removed manually and the coding region was translated to amino acid sequences and compared with the CHS of *M. sativa.* The comparative analysis showed remarkable substitutions of amino acid residues in *A. calcarata*: Thr (197) to Ser, Ser (338) to Gln, Ileu (254) to Val, when compared to typical CHSs. The numbering and type of amino acid residues is based on the amino acid sequence of *M. sativa* CHS*.* These changes observed on *A. calcarata* PKS can be taken as an indication of function(s) other than typical chalcone formation. The presence of the unusual 93 bp intron in *A. calcarata* may be of significance to the evolution of the PKS gene family in Zingiberaceae. All CHS genes studied so far contained an intron at a conserved position, but in *Antirrhinum majus* a second intron was found to be present in the second exon (Sommer and Saedler, 1986). Very recently, CHS superfamily members with two introns or without an intron have been reported from *Physcomitrella patens* (Jiang *et al.*, 2006). Yet, the current result is the first report of an intron in the second exon of PKS from Zingiberaceae. A recent report of a novel Type III polyketide synthase encoded by a three-intron gene in *Polygonum cuspidatum* (Ma *et al.*, 2009) makes the current result even more interesting.

The phylogenetic analysis of AcPKS with other PKS sequences showed separate clustering, distinct from typical CHS/PKSs and along with recently identified plant-specific nonchalcone forming PKSs (Figure 2). The separate clustering of the sequence may be an indication of its biochemical distinctness. The clustering of AcPKS with the recently characterized PKSs of the curcuminoid synthase group also supports its this view (Katsuyama *et al.*, 2007, Katsuyama *et al.*, 2009a, and Katsuyama *et al.*, 2009b). All the nonchalcone forming PKSs form a separate cluster, different from the typical CHSs. The biosynthetic role of nonchalcone forming PKSs is well studied and shown to play key role in building the structural skeleton of medicinal natural products, and some of them may even form unnatural compounds (Abe *et al.*, 2002). In the case of *Rheum palmatum*, a phylogenetic analysis including four members of the PKS superfamily generated separate clusters for CHSs and non CHSs. Such separate clustering was also reflected in the biochemical activities (Abe *et al.*, 2005).

Most of the genes coding for the biosynthetic machinery of plant secondary metabolism are encoded by small families of genes originated through duplication over evolutionary time (Durbin *et al.*, 2000). Type III polyketide synthase or chalcone synthase occurs in most plants as multigene families (Radhakrishnan and Soniya, 2009). In legumes, it forms multigene family of 6 to 12 members (Tuteja *et al.*, 2004; Matsumura *et al.*, 2005). The genomic distribution of the PKS superfamily in medicinal plants is yet little investigated and their analysis may result in novel insights into the functional and evolutionary features of PKSs. Sequence analysis of the second exon of *A. calcarata* PKS identified in this study shows the unusual occurrence of an intron that can be considered as a novelty. The sequence identified in this study opens a perspective for further molecular exploration of the PKS family in *A. calcarata.*

**Figure 1:**
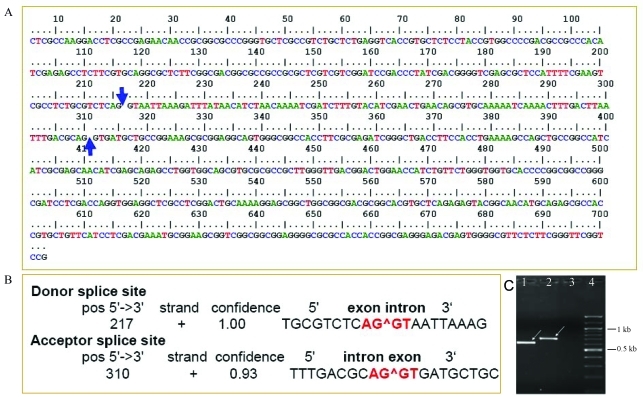
A. Intron prediction for the *Alpinia calcarata* PKS sequence carried out at the NetPlantGene web server. The arrows represent putative splice sites. B. A donor splice site predicted at 217 bp and an acceptor splice at the 310 bp position. C. Amplification of a core fragment of the *Alpinia calcarata* PKS gene. Lanes (1) PCR product obtained from genomic DNA of *Zingiber officinale*, used as positive control, (2) PCR product obtained from genomic DNA of *Alpinia calcarata*, (3) negative control, and (4) 100 bp DNA ladder.

**Figure 2:**
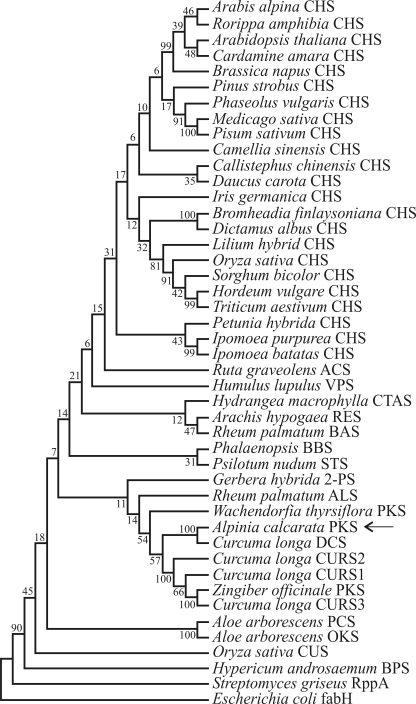
Phylogenetic analysis of AcPKS with other members of PKS superfamily, using the NJ method implemented in MEGA 3 and 1000 bootstrap replicates. The AcPKS identified in this study is marked by an arrow. It forms a cluster with members of the nonchalcone forming PKSs.  Abbreviations: CHS – chalcone synthase; STS – stilbene synthase; 2-PS – 2-pyrone synthase; ACS – acridone synthase; ALS – aleosine synthase; BBS – bibenzyl synthase; CTAS - 4-coumaroyltriacetic acid synthase; BAS – benzalacetone synthase; VPS – valerophenone synthase; PKS – polyketide synthase; RES – resveratrol synthase; BPS – benzophenone synthase; PCS – pentaketide chromane synthase; OKS – octaketide chromane synthase; RppA – red-brown pigment producing enzyme; FABH – b-ketoacyl carrier protein synthase III (out group), CUS and CURS – curcuminoid synthase; DCS – diketide CoA synthase.
